# Response to Hartwig and Davies

**DOI:** 10.1093/ije/dyw252

**Published:** 2016-09-20

**Authors:** Jack Bowden, Stephen Burgess, George Davey Smith

**Affiliations:** ^1^MRC Integrative Epidemiology Unit, University of Bristol, Bristol, UK,; ^2^MRC Biostatistics Unit, Cambridge Institute of Public Health, Cambridge, UK and; ^3^Department of Public Health and Primary Care, University of Cambridge, Cambridge, UK

Thank you for the opportunity to respond to the thoughtful and timely letter by Hartwig and Davies.^1^ They do indeed raise a very important practical issue with the implementation of MR-Egger regression in the single-sample setting, or with the use of so called `internal’ weights, namely weak instrument bias. They are right to point out the unsatisfactory nature of our analysis of the height data in our original publication,^2^ and that our use of weak instruments had the likely effect of biasing the MR-Egger estimate towards that of the observational association. Their re-analysis of these data with external weights appears to provide a much more satisfactory answer and, when such weights are available, it is both a simple and an attractive way to circumvent the problem.

Although previous simulation studies have highlighted this fact, further research is needed to completely understand the issue of weak instrument bias for MR-Egger in the single-sample context. What is clear however, is that the standard notion of instrument strength, as quantified by the F statistic, cannot naively be applied to estimate the magnitude of this bias; new (or at least newly borrowed) theory is required. Before covering initial progress in this vein, we now briefly discuss related (and more mature) work in the two-sample context.

## Recent work on weak instrument bias in the two-sample context

A strength of MR-Egger regression, along with the weighted median^3^ and inverse-variance weighted (IVW)^4^ methods, is that they do not require gene, exposure and outcome data on a single sample of subjects at the individual level. MR-Egger regression can be implemented with only summary data estimates of the single nucleotide polymorphism (SNP)-exposure and SNP-outcome associations, making it most natural to use within the two-sample summary data context. This is becoming an increasingly popular way of performing Mendelian randomization investigations and, in recent work to appear in this journal,^5^ we extensively explore the issue of weak instrument bias for MR-Egger in this simpler setting. We show that weak instruments (with small F statistics) have the effect of inducing regression dilution bias into the MR-Egger estimate, thus shrinking its value towards the null (although such bias does not inflate Type 1 error rates when testing for a causal effect). This same phenomenon has also been shown for the IVW estimate.^6^^,^^7^ However, whereas the magnitude of the attenuation in the IVW estimate can be gauged from the instrument’s F statistics (e.g. a mean F of 20 would indicate an approximate dilution of 1/20 = 5%), this will almost certainly underestimate the dilution for MR-Egger. A new statistic - which is a simple modification of Higgins’ $I^{2}$ - can be used to quantify the dilution (we call it $I_{GX}^{2}$). It lies between 0 and 100%, and an $I_{GX}^{2}$of 95% would indicate an expected dilution of 5%. We also describe how the established method of simulation extrapolation (SIMEX)^8^ can be used to calculate a bias-adjusted MR-Egger estimate to mitigate the effect of this dilution.

## Preliminary results and further work in the single-sample context.

The first author of this letter is currently conducting a theoretical investigation of the weak instrument bias of MR-Egger regression in the single-sample setting, with particular focus on comparing its performance to that of the IVW estimate. Initial results highlight the following: (i) when all genetic instruments are valid, both MR-Egger and IVW estimates are consistent, but the MR-Egger estimate will always be more strongly affected by weak instrument bias (towards the observational association) than the IVW estimate; and (ii) when some or all of the genetic instruments are invalid due to directional pleiotropy, but the sample covariance of the pleiotropy and instrument strength terms is zero (e.g. the InSIDE assumption in Bowden *et al.*^2^ holds), then MR-Egger is a consistent estimate of the causal effect whereas the IVW estimate is asymptotically biased. However, the finite-sample bias of the IVW estimate may still be less than that of MR-Egger if the genetic variants are sufficiently weak.

Research is under way to investigate whether a suitable statistic, perhaps analogous to $I_{GX}^{2}$, can be derived to quantify the likely magnitude of dilution of MR-Egger regression in the single-sample setting. An obvious follow-on question will be whether the SIMEX approach outlined in this journal^5^ can also be transferred to this setting to yield a bias-adjusted estimate. This is not trivial because such an implementation is likely to require at least some a priori knowledge of the strength of correlation between the standard errors of the SNP-exposure and SNP-outcome association estimates due to shared confounding (in the two-sample context we assume *a priori* this correlation is zero). Theoretical work on this topic already exists^9^ and will no doubt serve as a useful starting point.

Even in cases which appear superficially to be two-sample settings, sample overlap may mean that the bias due to weak instruments is more similar to the single-sample setting. For example, around 71% of participants are shared between the Genetic Investigation of Anthropometric Traits (GIANT) consortium^10^ and the Global Lipids Genetics Consortium (GLGC).^11^ For the IVW method, the direction of weak instrument bias varies linearly as the proportion of sample overlap increases from the two-sample setting (where bias is in the direction of the null) to the single-sample setting (where bias is in the direction of the observational association).^12^ Further work is needed to see if a similar pattern holds for the MR-Egger method.
